# Self-Consistent Field Modeling of Pulling a Test-Chain away from or Pushing It into a Polymer Adsorption Layer

**DOI:** 10.3390/polym12081684

**Published:** 2020-07-28

**Authors:** Fransicus A.M. Leermakers

**Affiliations:** Physical Chemistry and Soft Matter, Wageningen University and Research, Stippeneng 4, 6708 WE Wageningen, The Netherlands; frans.leermakers@wur.nl

**Keywords:** force spectroscopy of polymer desorption, coil-to-flower phase transition, polymers at interfaces

## Abstract

We consider single chain force measurements to unravel characteristics of polymers at interfaces and to determine parameters that control adsorption or probe layer characteristics that are difficult to access otherwise. The idea is to have at the tip of an atomic force microscope (AFM), a probe chain and measure its behaviour near interfaces by pushing it to, or pulling it away from it. The self-consistent field modeling of this reveals that in the pulling mode—i.e., when the chain has an affinity for the surface—a typically inhomogeneous flower-like conformation forms with an adsorbed ’pancake’ and a stretched stem (tether) from the surface to the tip of the AFM. When about half the segments is in the tether it snaps loose in a first-order like fashion. The critical distance of the end-point from the surface and the critical force are experimentally accessible. Details of this transition depend on the surrounding of the test chain. Inversely, and this opens up many possibilities, the test chain reports about its surroundings. Our focus is on the classical case of homopolymers at interfaces. Pulling experiments may reveal the adsorption strength, the (average) chain length and/or the polymer concentration of the freely dispersed/adsorbed polymers. When the test-chain is non-adsorbing we envision that pushing this test-chain into the adsorption layer reports about various layer characteristics such as the layer thickness and (local) density. Moreover, when the test-chain has a length longer than the entanglement length, we can imagine that non-trivial dynamical properties of loops and tails may be scrutinised.

## 1. Introduction

Polymers at interfaces is a classical field of science with a rich history [1]. The self-consistent field (SCF) theory for polymers at interfaces is well-developed and agrees in a qualitative manner with the scaling picture of the layer [1,2,3]. Indeed the de Gennes picture of a proximal, a central and a distal structure of the adsorbed layer is by now well established [4]. Only in the inner part of the central region the SCF theory predicts for good solvents and strong adsorption $\varphi \left(z\right)\propto z^{-2}$, whereas the scaling picture gives $\varphi \left(z\right)\propto z^{-4/3}$ [5]. The discrepancy is attributed to a failure of SCF to account for (lateral) excluded volume correlations. In the outer part of the central region it was recently shown that SCF is consistent with the de Gennes picture as in the SCF approach the long tails feel (arguably artificially) a nearby escape transition: the long tails form a tether trying to escape from the central region. This was argued to be enough to find the −4/3 power-law coefficient in the density profile.

Focusing on generic features of polymers at interfaces is for many practical systems rather academic. To do quantitative predictions for polymers at interfaces requires knowledge of the governing interaction parameters, such as the solvent strength, which in the Flory–Huggins picture is represented by $\chi \equiv \chi_{AW}$, where A is the polymer segment and W is the solvent, and the corresponding interaction with the surface, S, $\chi_{AS}$. The solvent quality parameters can be estimated in various ways and reasonable estimates are usually available. However, the interactions with a particular surface are typically less well documented [6,7,8].

With respect to finding out what the value is for the adsorption energy, it has been suggested earlier that one may use a displacer technique to ‘push’ the polymer off the surface [6,7,8]. The amount of displacer needed to force the polymer from a substrate is a measure for the strength of adsorption. Adsorption may be measured e.g., by an optical technique [7] or by some chromatographic method. When large amounts of the displacer are needed there is also an influence on the solvent quality. Complementary methods to obtain similar information are therefore welcomed.

Recent developments in single chain experiments open new venues to find information on polymer adsorption layers [9]. In particular our interest is drawn to a classical problem of pulling an adsorbed chain away from the surface by pulling on one of its ends [10]. From a theoretical perspective, Eisenriegler focused on isolated chains and argued that this process leads to flower-like inhomogeneous conformations of the chain and the transition from bound to the surface to unbound is first-order like [11,12].

**Figure 1:**
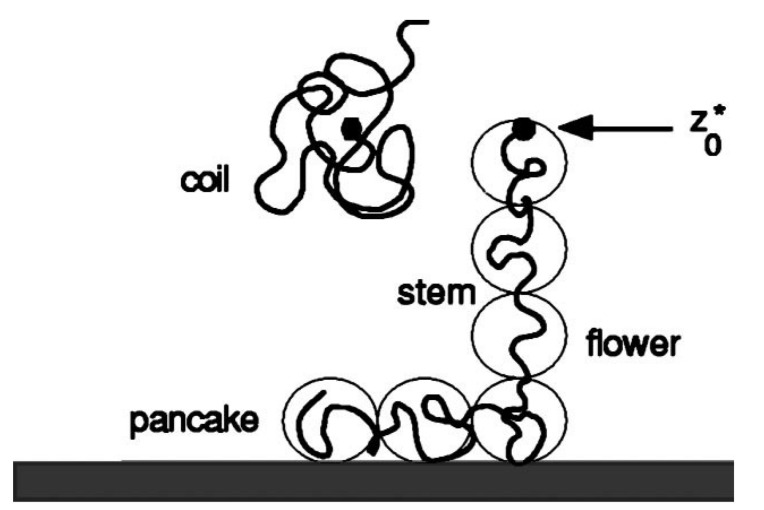
Illustration of the coil-to-flower transition for an adsorbed chain pulled by one end away from an adsorbing surface. The flower is composed of *m* adsorbed segments (pancake) and N−m stretched segments (stem). The spheres are illustrative for the respective blobs, near the surface they have the size $\xi_{a}=1/c$ (*c* is the de Gennes adsorption parameter, and in the tether $\xi_{s}=1/f$ (*f* is the pulling force). $z_{0}$ is the position of the end of the chain. At the first-order transition the position is denoted by $z_{0}^{*}$. At that point the number of adsorption blobs equals the number of stretching blobs and the free energy of the coil equals that of the flower.

The latter can already be seen from a simple Flory-type argument. We consider a chain with *N* segments, which is put in a flower-like conformation with *m* segments in contact with the surface, forming a ‘pancake’ of adsorption blobs of size $\xi_{a}$ and N−m segments in the tether forming a string of stretching blobs of size $\xi_{s}$. Let the end of the tether be located at a distance $z=z_{0}$ from the surface. For the Gaussian chains one can write a free energy of the inhomogeneous conformation as (cf Figure 1) [13,14,15]:

(1) $$F\left(m\right)=-m\frac{c^{2}}{6}+\frac{3}{2}\left(\frac{z_{0}^{2}}{N-m}\right)$$

In this equation the first term gives the adsorption contribution of the part next to the surface (the pancake) and the second term is due to the stretching of the chain in the tether. The *c* is the de Gennes adsorption parameter [4,16]. It is negative for adsorption and positive for depletion, while c=0 corresponds to the critical value. For adsorption $\xi_{a}=\left|1/c\right|$ is the layer thickness of the pancake. Optimization of F(m) with respect to *m* gives $z_{0}=\frac{\left|c\right|}{3}\left(N-m\right)$. Inserting this in Equation (1) shows that $F=\frac{c^{2}}{6}\left(N-2m\right)$. When the chain is far from the surface, its conformation relaxes to the Gaussian size for which the free energy is zero (by reference). Hence the chain snaps loose when N=2m, that is when half the segments is in the pancake and half in the tether (binodal condition). When the chain snaps loose, the end-point is at $z_{0}^{*}=\left|c\right|N/6$. At the transition the stretching blob $\xi_{s}=3\left(N-m\right)/z_{0}^{*}=1/\left|c\right|\equiv \xi_{a}$. For the excluded volume chain the qualitative picture remains [13].

The force to maintain the end of the chain at a given distance $z_{0}$ is given by $f=-\partial F\left(m\right)/\partial z_{0}$. A rather simple force-distance picture emerges. As long as the force exerted on the free end is below the pull-off value f<c the chain end remains near the surface, then at the pull-off force f=c the end is moved away from the surface where it snaps loose when the critical distance is passed and the force abruptly drops to zero. The Flory argument suggests that both the detachment distance and the detachment force are proportional to the adsorption strength. Hence both quantities behave in line.

On the Gaussian chain level we have the exact partition function of the adsorbed chain with an force exerted on one of its ends and the first-order phase transition when the chain snaps loose from the surface has been analyzed in great detail [13]. There are few works that go beyond the Gaussian chain level [17]. By far the majority of more exact results are generated by computer simulations [18,19,20,21,22,23].

The coil-to-flower transition which is sketched for the detachment of the adsorbed chain is discussed explicitly for isolated chains. In the presence of other chains the picture becomes more involved. Below we will use the self-consistent field theory to analyse the free energy as a function of distance curves in such more complicated cases. It is shown that the force distance curves are informative about the surroundings of the chain that is pulled away from the surface. As this force distance curve can in principle be measured by AFM, we argue that a diagnostic tool presents itself: we can learn with these measurements about various details of the physical characteristics of the macromolecules that compete with the probe-chain for the adsorbing surface.

We foresee two types of application for such diagnostic tool. In a pulling test we may learn e.g., about the adsorption strength by which the competing macromolecules adhere to the surface. This mode is effective when the test chain adsorbs preferentially at the surface. Inversely when the freely dispersed/adsorbed chains adsorb preferentially, we can do a pushing test. Pushing the test chain in a thick adsorption layer may reveal details about this adsorption layer. For example we may establish the overall thickness, the (local) density etcetera. In particular the latter type of experiments may be complementary to scattering experiments (targeted to find polymer distributions) [1], or hydrodynamic experiments (targeted to find the thickness) [24].

The remainder of the paper is organised as follows. First we will briefly highlight the self-consistent field (SCF) theory and describe in particular the geometry in which the SCF calculations are performed. In the results section we will present a couple of case studies to illustrate what type of information can (at least in principle) be obtained. We understand that experimentally there are many intricacies to gather the suggested information by single chain force spectroscopy (AFM) measurements. In the discussion we will briefly elaborate on a few obvious issues, but also highlight the many opportunities. In the conclusion we summarize our ideas.

## 2. Self-Consistent Field Modeling

We use the self-consistent field protocol with the discretization scheme of Scheutjens and Fleer (SF-SCF) [1,2,5,25]. In this method there exists a free energy functional basically expressed in complementary distribution, the segment volume fractions φ(r) and the complementary segment potentials u(r). Such pair is present for each segment type X=A,B,… in the system. The r represent discrete coordinates. Here and below we will use a two-gradient cylindrical coordinate system, with r=(r,z), with $r=1,\;2,\;\cdots ,\;M_{r}$ ‘sites’ in the radial and $z=1,2,\cdots ,M_{z}$ sites in the longitudinal direction; each site has a linear length *b*. At each coordinate (r,z) there are $L\left(r\right)=\pi (r^{2}-{\left(r-1\right)}^{2})=\pi \left(2r+1\right)$ lattice sites. The use of volume fractions at such coordinate implies a mean-field approximation. Density fluctuations in the radial direction are ignored. A solid surface is placed at z=0. At all other boundaries we will implement mirror-like boundary conditions (Neumann boundary conditions). All linear lengths are normalized by *b*.

The SF-SCF method adopts the Flory–Huggins equations of state [26]. The interactions are evaluated using the Bragg-Williams approximation generalised for inhomogeneous systems. The contact interactions are parameterized by Flory–Huggins exchange interaction parameters $\chi_{XY}=\frac{Z}{2k_{B}T}\left[2U_{XY}-U_{XX}-U_{YY}\right]$ as usual where *Z* is the lattice coordination number (here we use a simple cubic lattice, hence Z=6), $k_{B}T$ is the thermal energy and all energies as well as the potentials are normalized with it. Similarly as in Flory–Huggins the system is taken to be incompressible, which means that the sum over all volume fractions add up to unity: $\sum_{X}\varphi_{X}\left(r\right)=1$. This condition is enforced by a set of Lagrange parameters α(r) [25].

In the system we have polymers that are composed of segments. The segments in the each chain *i* are numbered $s=1,\;2,\;\cdots ,\;N_{i}$. The segments are assumed to have a unit length *b* as well. One segment fits nicely on a given lattice site and segments can only take positions specified by the above coordinate system. The chain model that is embraced by the SF-SCF method is the lattice freely jointed chain model. In such a model neighboring segments along the chain will occupy neighboring lattice sites. Longer ranged (along the chain) correlations are ignored (Markov chain).

The mentioned free energy in terms of segment densities and segment potentials is extremized with the help of the Lagrange method which implements the compressibility constraint. This extremisation translates in a minimization with respect to the densities, but a maximalisation with respect to the potentials and the Lagrange field. The result of this extremisation leads to the well known self-consistent field equation.

The optimisation with respect to the densities give an equation for the segment potentials:

(2) $$u_{X}\left(r,z\right)=\alpha \left(x,z\right)+\sum_{Y}\left(\langle \varphi_{Y}\left(r,z\right)\rangle -\varphi_{Y}^{b}\right)$$

Here the angular brackets give a local averaging:

(3) $$\begin{array}{c} \langle \varphi \left(r,z\right)\rangle =\frac{1}{6}\left[\frac{A(r-1)}{L(r)}\varphi \left(r-1,z\right)+\frac{A(r)}{L(r)}\varphi \left(r+1,z\right)+2\varphi \left(r,z\right)+\varphi \left(r,z-1\right)+\varphi \left(r,z+1\right)\right] \end{array}$$

with A(r)=2πr, is the ‘area’ per unit length associated to the ‘layer’ coordinate *r*. In the potentials the bulk volume fraction $\varphi_{Y}^{b}$ is introduced to enforce that the segment potentials are zero in the bulk. Typically, we may take it that bulk concentration is specified as an input.

The optimisation of the free energy with respect to the potentials leads to a rule how to compute the volume fractions. In the freely jointed chain model this route requires the evaluation of the chain partition functions for specified values of the segment potentials. From these partition functions the densities follow naturally. The procedure is implemented using an efficient propagator formalism which is well known in the polymers at interfaces community. For completeness we briefly specify this protocol. The volume fractions are computed by a composition law. For the solvent (i=0), for the tethered (probe) chain (i=1) and the freely dispersed chains (i=2) we have respectively

(4) $$\begin{array}{ccc} \varphi_{0}\left(r,z\right) & = & C_{0}G_{0}\left(r,z,1\right)\\ \varphi_{1}\left(r,z,s\right) & = & C_{1}\frac{G_{1}\left(r,z,s|1,z_{0},1\right)G_{1}\left(r,z,s|N\right)}{G_{1}\left(r,z,s\right)}\\ \varphi_{2}\left(r,z,s\right) & = & C_{2}\frac{G_{2}\left(r,z,s|1\right)G_{2}\left(r,z,s|N\right)}{G_{2}\left(r,z,s\right)} \end{array}$$

where $G_{i}\left(r,z,s\right)=\exp-u_{i}\left(r,z,s\right)$ is the free segment distribution function for molecule type *i*. The potential that features in this Boltzmann-like function is needed for segment *s* of molecule *i*. From the input we know the segment type of this segment, that is we know the full architectural information of all chains: $\delta_{i,s}^{X}=1$ when segment *s* of molecule *i* is of type *X* and zero otherwise. Hence $u_{i}\left(r,z,s\right)=\sum_{X}\delta_{i,s}^{X}u_{X}\left(r,z\right)$. The composition law (4) features end-point distributions $G_{1}\left(r,z,s|1,z_{0},1\right)$, $G_{i}\left(r,z,s|1\right)$ and $G_{i}\left(r,z,s|N\right)$. These contain the combined statistical weights of all freely jointed chain walks that end with segment *s* at coordinate (r,z), starting with segment s=1 at $\left(r,z\right)=\left(1,z_{0}\right)$, at any position of segment s=1 and any position of s=N, respectively. Fragments that contain just one segment obey to $G_{1}\left(r,z,1|1,z_{0},1\right)=G_{1}\left(r,z_{0},1\right)$, $G_{i}\left(r,z,1|1\right)=G_{i}\left(r,z,1\right)$ and $G_{i}\left(r,z,N|N\right)=G_{i}\left(r,z,N\right)$. Longer end-point distributions are found from shorter ones:

(5) $$\begin{array}{ccc} G_{1}\left(r,z,s|1,z_{0},1\right) & = & G_{1}\left(r,z,s\right)\langle G_{1}\left(r,z,s-1|1,z_{0},1\right)\rangle \\ G_{i}\left(r,z,s|1\right) & = & G_{i}\left(r,z,s\right)\langle G_{i}\left(r,z,s-1|1\right)\rangle \\ G_{i}\left(r,z,s|N\right) & = & G_{i}\left(r,z,s\right)\langle G_{i}\left(r,z,s+1|N\right)\rangle \end{array}$$

The normalization *C* of the composition law (4) is dependent on the situation. For the probe-chain we compute the single chain partition function by $q_{1}=G_{1}\left(1,z_{0},1|N\right)$ and normalize the densities by $C_{1}=1/q_{1}$. For the unconstrained molecules the bulk concentration $\varphi_{i}^{b}$ is the input and $C_{i}=\varphi_{i}^{b}/N_{i}$.

The volume fraction for a specific segment type *X* is found by

(6) $$\varphi_{X}\left(r,z\right)=\sum_{i}\sum_{s}\delta_{i,s}^{X}\varphi_{i}\left(r,z,s\right)$$

and similarly the bulk concentration for segment type *X* follows from $\varphi_{X}^{b}=\sum_{i}\sum_{s}\delta_{i,s}^{X}\varphi_{i}^{b}$.

From the above it is seen that we can compute the potentials when we know the densities (and the Lagrange parameters) but we need the potentials to compute the densities. This problem can in general only be solved numerically. Basically we go iteratively from potentials to densities and back again to the potentials until a fixed point is reached. Meanwhile we update the Lagrange field updated with $\alpha \left(r,z\right)\leftarrow \alpha \left(r,z\right)+(\sum_{X}\left(\varphi_{X}\left(r,z\right)-1\right)$, until also this quantity arrives at a fixed point. In practise a more sophisticated iteration scheme is used which results in seven significant digits in order 100 iterations [25].

In the system we typically have one (constrained) probe/test chain, while all other molecules (including a monomeric solvent) are unconstrained. Hence we have a partial open system, and the characteristic function for this is $F^{\mathrm{po}}=F-\sum_{j}\mu_{j}n_{j}$ wherein the summation over all molecules *j* is over the unconstrained molecules. The number of molecules $n_{j}=\sum_{r}L\left(r\right)\sum_{z}\sum_{s=1}^{N_{j}}\varphi_{j}\left(r,z,s\right)/N_{j}$. For the probe-chain (test-chain) this sum leads exactly to unity. The chemical potential $\mu_{j}$ is computed from the information in the homogeneous bulk for which the Flory-Huggins model applies. The general equation is

(7) $$\mu_{j}=\ln\varphi_{j}^{b}+1-N_{j}\sum_{k}\frac{\varphi_{k}^{b}}{N_{k}}-\frac{N_{j}}{2}\sum_{X}\sum_{Y}\left(\varphi_{X}^{b}-\frac{N_{j}^{X}}{N_{j}}\right)\chi_{XY}\left(\varphi_{Y}^{b}-\frac{N_{j}^{Y}}{N_{j}}\right)$$

where $N_{j}^{X}$ is the number of segments *X* in molecule *j*. Finally the free energy (Helmholtz energy) is given by

(8) $$F=-\ln Q-\varphi \cdot u+F^{\mathrm{int}}$$

In the mean-field approximation the system partition function can be decomposed $Q=\Pi_{i}q_{i}^{n_{i}}/n_{i}!$, further, with *q* is the single chain partition function; $\varphi \cdot u=\sum_{r}L\left(r\right)\sum_{z}\sum_{X}\varphi_{X}\left(r,z\right)u_{X}\left(r,z\right)$ and finally the interaction term $F^{\mathrm{int}}=\sum_{r}L\left(r\right)\sum_{z}\sum_{X}\sum_{Y}\chi_{XY}\varphi_{X}\left(r,z\right)\langle \varphi_{Y}\left(r,z\right)\rangle$.

Our focus is on situation that the probe chain is pinned at coordinates $\left(1,z=z_{0}\right)$, and we obtain as a result of the SCF equations the free energy $F(z_{0})$. Below we will subsequently normalize this free energy such that it is zero when the probe chain is pulled completely away from the surface (that is when z→∞). One can obtain the force by $f=-\partial F/\partial z_{0}$.

The default system size we set $M_{r}=25$ and $M_{z}=200$, and the probe chain i=1 has a length $N_{1}=100$ and the segment type is referred to by A. There is a monomeric solvent with segment type *W*. The default solvent quality is $\chi_{AW}=0$ (both for the probe chain as well as other polymers in the system). Some calculations were done for the theta solvent conditions $\chi_{AW}=\chi_{BW}=0.5$. The surface is denoted by segment type *S* and exists at all positions of z<1. For the adsorption energy the interaction of the solvent to the surface is the reference $\chi_{SW}=0$. A negative value for $\chi_{AS}$ implies attraction. When a second component is in the system we will assume that it has segments of type *B*. Without mentioned otherwise we will set the solvent quality for *B* identical to that of *A*, hence $\chi_{AW}=\chi_{BW}$.

In the Flory model we have made use of de De Gennes adsorption parameter *c*, whereas the lattice model has the Flory–Huggins parameter $\chi_{AS}$ (with the Ansatz that $\chi_{SW}=0$). Mapping of these models proved that these parameters are to a good approximation related as [27,28]

(9) $$-\chi_{AS}=-c^{2}+6\ln\left(\frac{4+e^{c}}{6}\right)$$

In the range of moderately strong adsorption $-6<\chi_{AS}<-2$ we have to a good approximation a proportionality of $\chi_{AS}$ with *c*.

## 3. Results

Let us first discuss the classical $F(z_{0})$ curve for our default system in the absence of additional polymers, that is, the isolated test chain. We selected good solvent condition χ=0.

Depending on the strength of the adsorption energy $\chi_{AS}<-1$ the test/probe chain tried to adsorb with its segments on the substrate *S*. We specified the distance from the surface $z_{0}$ of one of the ends and recorded the free energy *F* as a function of $z_{0}$ in such a way that *F* was normalized to zero for large values of $z_{0}$ where the chain completely detached from the surface.

**Figure 2:**
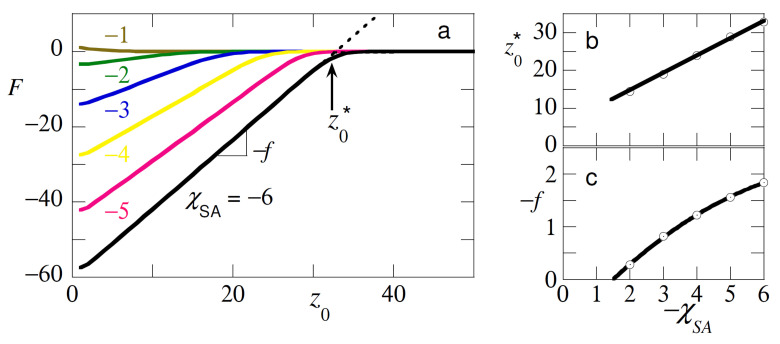
(**a**) Free energy *F* in units of $k_{B}T$ of a test chain with N=100 segments adsorbed on an impenetrable surface with adsorption strength $\chi {}_{S}A$ as indicted, as a function of the distance $z_{0}$ of one of the ends above the surface. Both, the slope which is related to the ‘retraction’ force −f and $z_{0}^{*}$, the distance where the chain ‘snaps’ loose, are indicated. (**b**) The detachment distance $z_{0}^{*}$ and (**c**) the detachment force *f* (in units of $k_{B}T/b$) as a function of the adsorption energy $\chi_{SA}$. Conditions identical to a.

As can be seen from Figure 2a the expected attractive interaction curves were observed. In experiments one was able to measure the force $f=-\partial F/\partial z_{0}$, which was needed to maintain the end at height $z_{0}$. For the adsorption regime this force was negative. Another quantity that could be measured experimentally was the detachment height $z_{0}^{*}$. In Figure 2b we present the detachment height and in Figure 2c the force. As expected from the Flory analysis presented above both the detachment height as well as the force decreased linearly upon decreasing the strength of adsorption.

The SF-SCF calculations deviated from the Flory analysis in two fundamental ways. Firstly, the chain model was the FJC and not the Gaussian chain. The difference showed up when the extension of the tether was on the order of its length (i.e., it was seriously noticed when the extension exceeded half its length). Then finite extensibility kicked in for the FJC model, but not for the Gaussian chain. Secondly, on the Flory-level excluded-volume effects were accounted for in the SCF model but not for the Gaussian approach. The test chain, for example, on the tip felt its own segments and swell accordingly. The coil size approached $R\propto N^{3/5}$. For these two reasons we could not quantitatively compare the SF-SCF results to the Flory-picture sketched above. We refrain from a detailed comparison with the Flory model and just mention that qualitatively the results matched. For example, we see, as predicted by the Flory-analysis that the force dependence and the detachment dependence behaved similarly with changes in the adsorption energy.

Inspection of Figure 2b shows that the detachment height scaled as expected linearly with the $\chi_{AS}$. When this height fell below the coil size *R* we were in the critical region and then the determination of this height became troublesome because the linearity of the interaction curves was lost. This is the reason why we here chose not to detail results for the critical region.

The force that was required to detach the chain from the surface (cf. Figure 2c) decreased with decreasing adsorption energy. Again, a close to linear dependence was found albeit that for large $\chi_{SA}$-values the force tended to level off slightly. Inspection of the force curve indicated that the attractive force vanished when $\chi_{SA}\approx -1$. This signaled the critical adsorption energy where the chain lost its adsorption strength for the surface. Close inspection of Figure 2a revealed a slight repulsion. This repulsive force was due to the compression of the coil against the non-adsorbing surface.

The results of Figure 2 are in principle well documented [13,14,15] and therefore we do without a more detailed analysis. Instead our focus is on the idea that the $F(z_{0})$-curves in the presence of macromolecules that compete for the surface, will report on the competition between these two chain types. By recording such curves we may obtain data that is welcomed in a systematic survey of polymers at interfaces. Below we will first consider the pulling and then the pushing regime.

### 3.1. The Pulling Regime

In this section we consider the case that the test/probe chain has a length $N_{A}=100$. It is further taken that it has a strong affinity to the substrate $\chi_{AS}=-6$. We are going to analyse the detachment characteristics of this chain in the presence of another type homopolymer in the system. This chain is present with a fixed bulk concentration $\varphi_{B}^{b}=10^{-3}$ which is in the dilute regime. We assume athermal solvent conditions for both chain types χ=0. Let us first consider the case that $N_{B}=N_{A}$, that is that the probe chain has the same length as the free chains.

**Figure 3:**
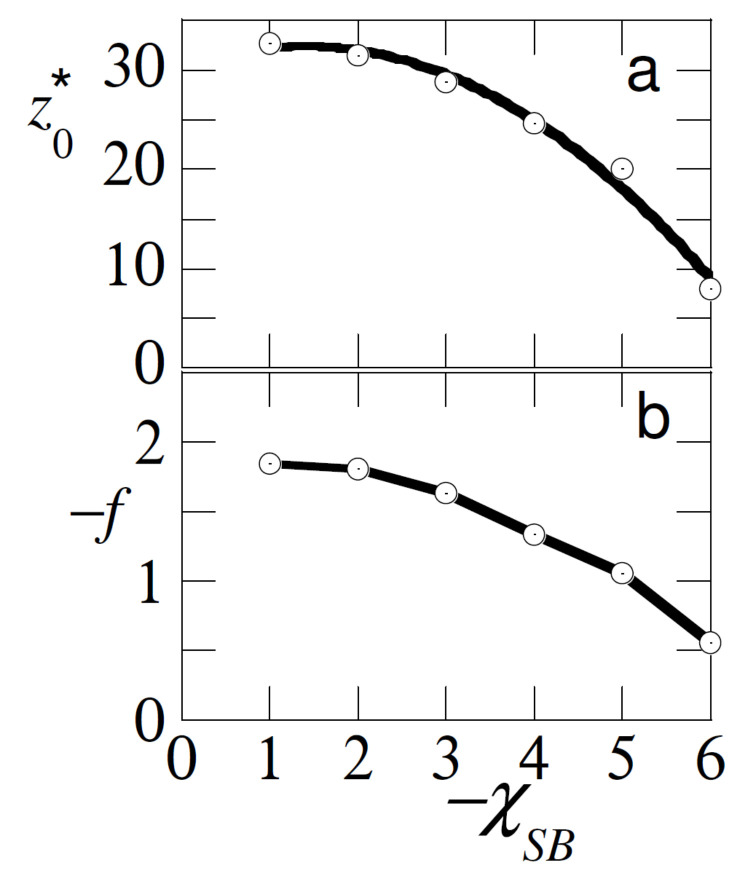
(**a**) The detachment distance $z_{0}^{*}$, (**b**) the detachment force −f in units of $K_{B}T/b$ of the test chain (A) as a function of the adsorption energy of the free chains (B) $\chi_{SB}$ in the system. Parameters $N_{A}=N_{B}=100$, $\varphi_{B}^{b}=10^{-3}$, $\chi_{AS}=-6$ and solvent quality is good χ=0.

In Figure 3 we have collected the detachment characteristics of the probe chain. When the adsorption strength of the free chains was identical to that of the probe chain $\chi_{SB}=\chi_{SA}=-6$, it is evident that the probe was displaced from the surface with a vanishing small displacement force and a minute displacement distance. It is evident that we were only just in the ’pulling regime’. As soon as $-\chi_{SB}<-\chi_{SA}$, the probe was preferentially at the surface. In this case $\Delta \chi_{S}=\chi_{SA}-\chi_{SB}<0$ was the ’effective’ adsorption strength of the probe. The larger this value the harder it was to pull the chain away from the surface, and thus the force increased, and the distance at which the probe detached $z_{0}^{*}$ increased. Obviously as soon as $\chi_{BS}>-1$, the free chains lost their interest in the surface and the unperturbed detachment distance and detachment force were recovered.

The results of Figure 3 may thus be used as an analytical tool, that is, by pulling the test chain from the surface, we may find out by which strength of adsorption the free chains are attached to the surface. Of course, we can only do so when the strength of adsorption of the test chain dominates, and other governing parameters (polymer concentration, chain length) are known.

**Figure 4:**
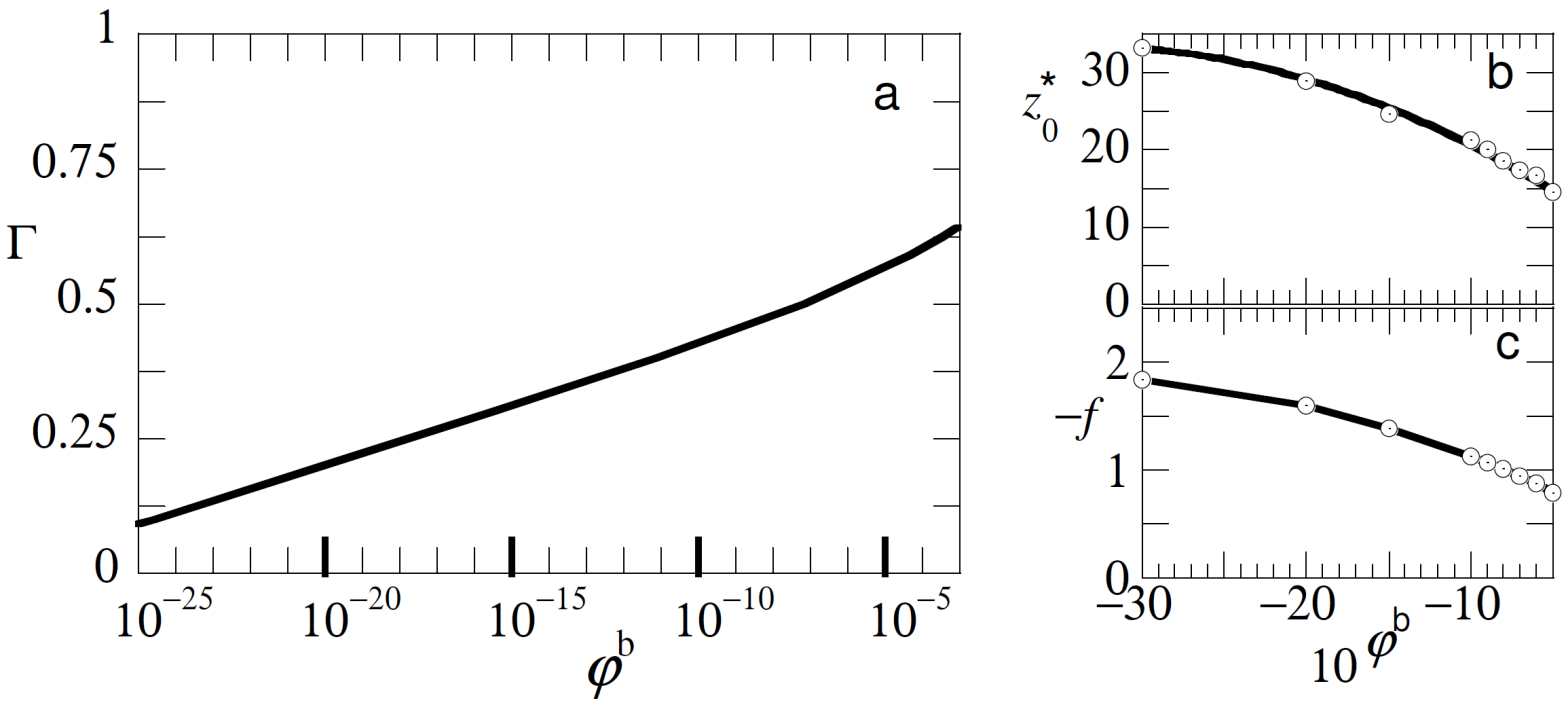
(**a**) Adsorption isotherm, that is $\Gamma \equiv \theta_{B}^{\mathrm{exc}}$ as a function of the bulk concentration $\varphi_{B}^{b}$ of the free chains in the solution in semi-logarithmic coordinates. (**b**) The detachment distance $z_{0}^{*}$ and (**c**) the detachment force, as a function of $10^{\varphi_{B}^{b}}$. Parameters: $N_{A}=N_{B}=100$, $\chi_{SA}=\chi_{SB}=-6$, χ=0.

In Figure 4 we show that, in addition, similar type of experiments may be informative about the concentration of free polymer in the solution. In Figure 4a we present the adsorption isotherm for free chains with length $N_{B}=100$ with an adsorption energy $\chi_{SB}=-6$ under good solvent conditions. The adsorbed amount Γ here is approximated by the excess amount $\theta_{B}^{\mathrm{exc}}=\sum_{z}\left(\varphi_{B}\left(z\right)-\varphi_{B}^{b}\right)$. The isotherm was of a high affinity type and in order to see also the initial part of the isotherm it was presented in semi-logarithmic coordinates. The characteristics of this isotherm are well known [1]. Typically, already well below the overlap concentration the adsorbed amount is close to the plateau value. To decrease the adsorbed amount at $\varphi_{B}^{b}=0.001$ by a factor of two, we needed to decrease the bulk concentration of the polymer to approximately $\varphi_{B}^{b}=10^{-14}$.

When we consider the detachment characteristics of the probe chain from adsorption layers that form for given bulk concentration of free polymers (cf Figure 4b,c), we see that both the detachment force and the detachment distance were noticeably affected by the bulk concentration up to concentrations of polymer (in this case) in the order of $10^{-30}$. In this limit the polymer layer was so-called starved, but still the probe chain was noticeably affected by the low fraction of adsorbed chains. This has everything to do with the cooperativity of adsorption and the high affinity type of the isotherm of course as the method does not directly measure the bulk concentration but the competition with the adsorbed chains on the surface.

Our conclusion is that, at least in principle, we may measure how the test-chain competes with the adsorbed chains even in the case the adsorption layer is starved. Indirectly one then gets information on the polymer concentration of the free chains. Again we can only determine these dependencies when the other characteristics (adsorption strength, chain lengths etc.) are known and the probe chain is preferentially adsorbed onto the surface over the free chains.

**Figure 5:**
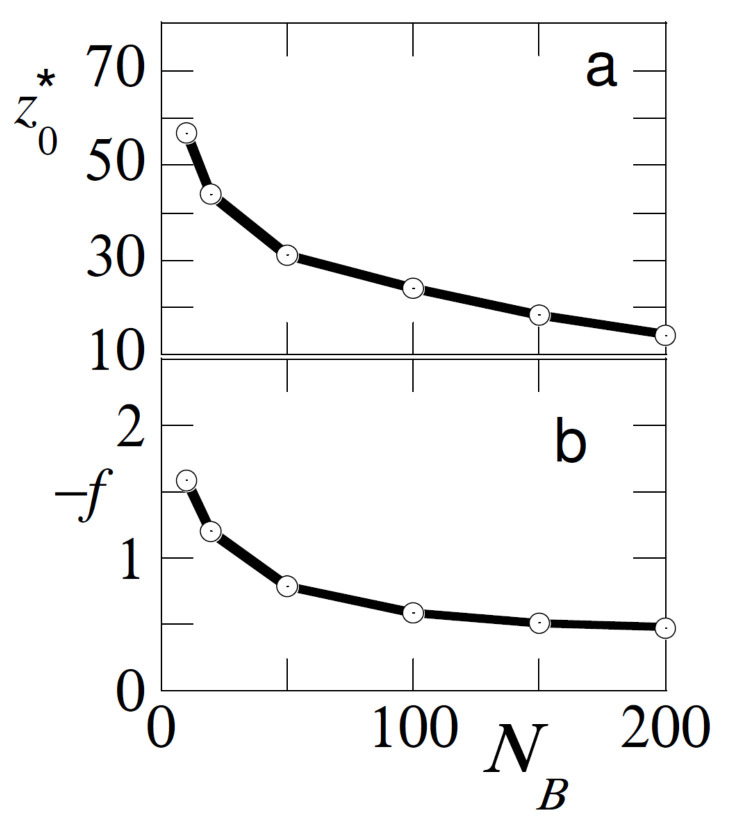
(**a**) Detachment distance $z_{0}^{*}$, and (**b**) detachment force *f* in units of $k_{B}T/b$ as a function of the length of the free chains $N_{B}$. Parameters: $N_{A}=200$, $\chi_{SA}=\chi_{SB}=-6$, $\varphi_{B}^{b}=10^{-3}$, χ=0.

In Figure 5 we investigate the effect of the chain length of the free chains for the case that the adsorption strength of the free and probe chain are similar $\chi_{SA}=\chi_{SB}=-6$. We return to the case that the bulk concentration of free polymer is $\varphi_{B}^{b}=10^{-3}$. In this result we increased the length of the probe chain to $N_{A}=200$. From classical adsorption theory [1] we know that long chains adsorb preferentially over short ones and therefor we find meaningful results for $N_{B}\leq 200$.

As is easily anticipated the shorter the free chains, the higher the dominance of adsorption of the probe chains and therefore it was harder to pull the probe from the surface (*f* is higher) and the larger the displacement distance $z_{0}^{*}$. Hence, the pulling experiments were also informative about the length of the free chains in the solution.

To sum up the above, it is clear that the detachment by pulling of the test chain is affected significantly by the adsorption strength, the chain length and the concentration of the free chains. If the adsorption strength is unknown, one should carefully control the length and the concentration of the free chains to unravel this strength of adsorption form the pulling experiments. On the other hand when the length of the chains is known and the adsorption energy is known, we have a means to determine the polymer concentration. Finally we can estimate the length of a polymer once the concentration and the adsorption energy are known.

### 3.2. Pushing Regime

In the ‘pushing’ regime we consider the case that the free chains that are in solution and readily adsorb onto the substrate have a higher affinity for the substrate than the probe. In this case the probe does not want to be near the surface. By forcing the free end to be in the vicinity of the surface, we may find information on the structure and density of the adsorbed layer. To illustrate this idea, we consider the free chains to have a length of $N_{B}=1000$ and adsorb strongly onto the substrate with an adsorption strength $\chi_{BS}=-6$. The polymer concentration was set to $10^{-3}$, which was close to, but below the overlap threshold.

**Figure 6:**
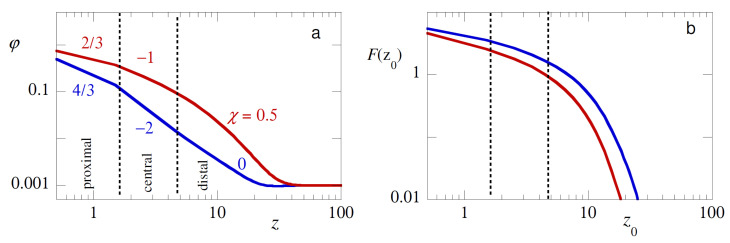
(**a**) The volume fraction profile $\varphi_{B}\left(z\right)$ in double logarithmic coordinates, for homopolymers with length $N_{B}=1000$, adsorbing from dilute solution $\varphi_{B}^{b}=10^{-3}$, with an adsorption strength $\chi_{SB}=-6$. The proximal, central and distal regions are indicated. The local slopes ∂logφ(z)/∂logz for the proximal and central regions are indicated. Red curve χ=0.5, Blue curve χ=0. (**b**) The corresponding $F(z_{0})$-curves.

As mentioned in the introduction, the segment density profile $\varphi_{B}\left(z\right)$ splits up in a proximal, a central and a distal region (cf. Figure 6a) [4]. In the case of strong adsorption the proximal zone became the size of the segments *b*. Here the density of polymer was melt-like. In the central region, the concentration was semi-dilute. As predicted by de Gennes the density had self-similar characteristics, that is, $\varphi \left(z\right)\propto z^{-\alpha }$, with α=−1 for theta solvent and α=−2 for (mean field) good solvents. The distal region was characterized by a logarithmic decay to the bulk density with a decay length proportional to the coil size. To date we still believe that the true coefficient in good solvent for the central region is −4/3, but strong numerical proof of this believe is not yet available. Experimental proves for the excluded volume scaling (−4/3) is also scarce [1,29,30].

In our view it is of considerable interest to probe the structure of the adsorbed layer by a pushing experiments. To this end we consider the case that the probe chain $N_{A}=100$ has no affinity for the substrate $\chi_{AS}=0$ and force the chain to penetrate the adsorption layer by fixing the position $z_{0}$ inside the adsorption layer. The free energy as a function of this distance $z_{0}$ is shown in Figure 6b again in double logarithmic coordinates. Admittedly the free energies in the pushing regime were way smaller than the typical values in the pulling regime, but in favourable cases we can easily imagine that one can pick up the corresponding pushing forces by AFM experiments.

Inspection of the pushing free energies we notice that for theta solvent the effects were smaller than in good solvent. This is expected because in theta solvent the second virial coefficient is zero and the tendency of the probe to ‘escape’ from the adsorption layer should be less intense. Comparison between the overall profile of the density and that of the free energy shows that at least the layer thickness of the adsorbed layer should be ‘visible’ by the pushing experiment. This is already relevant because in the SCF picture this distance is significant less than in the de Gennes picture. In the central region, the $F(z_{0})$-traces did not reproduce the exact power-law slopes. However, this information may in some way ‘hidden’ in the curves. By e.g., repeating for a given adsorption layer the pushing experiment with different lengths of the probe chain, we may possibly deconvolute the overall density profile. Work in this direction is of more than average interest.

## 4. Discussion

In principle we can systematically perform single molecule force experiments with an AFM [10]. These experiments necessary are done at a given movement speed of the AFM tip. The above analysis of the coil-to-flower-like transition corresponds to the ‘binodal’ conditions, whereas in dynamical experiments also the ‘spinodals’ are important [13]. By varying the speed by which the experiments are done one should be able to recover the binodal for each condition. That is, we need to extrapolate the result to the equilibrium limit, where the speed of the AFM tip no longer influences the detachment force/distance. Possibly to reach this limit one has to fine-tune the length of the probe chain, so that the characteristic time of the experiment is long compare to the Rouse time of the chain.

The idea that one can use pulling or pushing experiments by grafting a single chain onto the tip of an atomic force microscope has successfully be used already to unravel the strength of ionic interactions by Spruijt and coworkers [9]. These type of experiments were informative on the strength and type of ionic interactions for, e.g., complex coacervates. We now anticipate that similar approaches will be equally informative for the classical polymer adsorption layer.

We hasten to mention that one should not underestimate various experimental complications that may arise. We already discussed the dynamical aspects of the attachment/detachment of the probe chains. We may also understood that the anticipated results do not come from a single pulling or pushing experiment. The sampling of many force–distance curves invariably is part of the measuring protocol, but the developments in AFM equipment also allow us to do so [9].

The key idea is that single molecule force spectroscopy as suggested may give unique information on polymer adsorption layers which is hard to obtain otherwise. To date, key structural information on adsorption layers of homopolymers at interfaces is obtained from scattering experiments [1]. The advantage of the scattering technique is that it is an ensemble measurement and one gets averaged information. Indeed the first and in favourable cases the second moment of the adsorbed layer, that is, the adsorbed amount and the layer thickness, can be obtained, but more detailed information is very hard, perhaps impossible to get. Force measurements have the potential to find interesting characteristics that go beyond these scattering results. For example, when the probe chain has a sufficient length so that it might entangle with chains in the adsorption layer (this might happen when the chain is pushed in the proximal or central parts of the profile, one may find interesting dynamic forces related to the (de)entanglements processes.

We do understand that pushing the probe chain into and adsorption layer cannot be done without also inserting the AFM tip into this layer. The forces from this are automatically included and the necessary corrections for this may be nontrivial as well.

Alternatively, an anonymous reviewer suggested to use a test chain that is attracted to the adsorbed chains, similarly as done by Spruijt [9], and perform a pulling experiment to extract some information on the adsorbed chains. Especially in the regime that the strength of adsorption (of the adsorbed chains) exceeds the strength of the interaction between the chains, one may have hopes to find information on the local density of chains and the like. In any case, we hope that the current paper may initiate experiments in this direction.

## 5. Conclusions

We elaborate the idea that a probe chain attached to the tip of an AFM can be used to scrutinize properties of the adsorption layers of homopolymers at interfaces. This idea is studied using the self-consistent field calculations in which these experiments are mimicked. We envision that one can systematically try to find the strength of adsorption, the length of the freely adsorbing chains and/or the polymer concentration in solution, by analysing properties such as the retraction force and or the detachment distance of the probe from the adsorbing surface. Apart from such pulling experiments we also envision pushing experiments by which one forces a chain into an adsorption layer and measure the tendency of this chain to try to escape from it. Dynamic variants of such single molecule force spectroscopy experiments may also reveal local tendencies of the probe chain to entangle with the adsorbed chains and this gives insight in the extend of the concentrated and semi-dilute regions of the adsorption layer.

## References

[1] Fleer G., Cohen Stuart M., Scheutjens J., Cosgrove T., Vincent B. (1993). Polymers at Interfaces.

[2] Van der Linden C., Leermakers F. (1992). On the self-similar structure of adsorbed polymer layers: The dependence of the density profile on molecular weight and solution concentration. Macromolecules.

[3] Leermakers F. (2018). Self-Consistent Field Modeling of Homopolymers at Interfaces in the Long Chain Length Limit. Polym. Sci. Ser. C.

[4] De Gennes P.G. (1981). Polymer-solutions near an interface 1. Adsorption and depletion layers. Macromolecules.

[5] FAM L. (2020). Long tails with flower-like conformations undergo an escape transition in homopolymer adsorption layers. Macromolecules.

[6] Van der Beek G., Cohen Stuart M. (1988). The hydrodynamic thickness of adsorbed polymer layers measured by dynamic light-scattering -effects of polymer concentration and segmental binding strength. J. Physique.

[7] Van der Beek G., Cohen Stuart M., Fleer G. (1991). Polymer desorption by monomeric and polymeric displacers, as studied by attenuated total reflection FT-IR spectroscopy. Macromolecules.

[8] Dijt J., Cohen Stuart M., Fleer G. (1994). Surface exchange kinetics of chemically different polymers. Macromolecules.

[9] Spruijt E., van der Berg S., Cohen Stuart M., van der Gucht J. (2012). Direct measurement of the strength of single ionic bonds between hydrated charges. ACS Nano.

[10] Neuman K., Nagy A. (2008). Single-molecule force spectroscopy: Optical tweezers, magnetic tweezers and atomic force microscopy. Nat. Methods.

[11] Eisenriegler E., Kremer K., Binder K. (1982). Adsorption of polymer chains at surfaces: Scaling and Monte Carlo analyses. J. Chem. Phys..

[12] Eisenriegler E. (1983). Multicritical behavior of polymers near an adsorbing wall. Helvetica Phys. Acta.

[13] Skvortsov A., Klushin L., Leermakers F. (2002). Exactly solved polymer models with conformational escape transitions of a coil-to-flower type. Europhys. Lett..

[14] Skvortsov A., Klushin L., Fleer G., Leermakers F. (2009). Temperature effects in the mechanical desorption of an infinitely long lattice chain: Re-entrant phase diagrams. J. Chem. Phys..

[15] Skvortsov A., Klushin L., Fleer G., Leermakers F. (2010). Analytical theory of finite-size effects in mechanical desorption of a polymer chain. J. Chem. Phys..

[16] De Gennes P.G. (1982). Weight distribution of loops in a diffuse, adsorbed polymer layer. C.R. Acad. Sci. Paris II.

[17] Janse van Rensburg E., SWhittington S. (2013). Adsorbed self-avoiding walks subject to a force. J. Phys. A Math. Theor..

[18] Bhattachary S., Milchev A., Rostiashvili V., Vilgis T. (2009). Pulling an adsorbed polymer chain off a solid surface. Eur. Phys. J..

[19] Bhattachary S., Rostiashvili V., Milchev A., Vilgis T. (2009). Polymer desorption under pulling: A dichotomic phase transition. Phys. Rev..

[20] Bhattachary S., Rostiashvili V., Milchev A., Vilgis T. (2009). Forced-Induced Desorption of a Polymer Chain Adsorbed on an Attractive Surface: Theory and Computer Experiment. Macromolecules.

[21] Milchev A., Rostiashvili V., Bhattachary S., Vilgis T. (2010). Polymer desorption under pulling a 1st-order phase transition without phase coexistence. Phys. Proc..

[22] Paturej J., Dubbeldam J., Rostiashvili V., Milchev A., Vilgis T. (2014). Force spectroscopy of polymer desorption: Theory and molecular dynamics. Soft Matter..

[23] Paturej J., Erbas A., Milchev A., Rostiashvili V. (2014). Detachment of semiflexible polymer chains from a substrate: A molecular dynamics investigation. J. Chem. Phys..

[24] Cohen Stuart M., Mulder J. (1985). Adsorbed polymers in aqueous media the relation between zeta potential, layer thickness and ionic strength. Coll. Surf..

[25] Evers O., Scheutjens J., Fleer G. (1990). Statistical thermodynamics of block copolymer adsorption. 1. Formulation of the model and results for the adsorbed layer structure. Macromolecules.

[26] Flory P. (1953). Principles of Polymers Chemistry.

[27] Gorbunov A., Skvortsov A., van Male J., Fleer G. (2001). Mapping of continuum and lattice models for describing the adsorption of an ideal chain anchored to a planar surface. J. Chem. Phys..

[28] Fleer G., Svortsov A. (2012). Reconciling lattice and continuum models for polymers at interfaces. J. Chem. Phys..

[29] Aubray L., Cotton J. (1981). Self-similar structure of an adsorbed polymer layer comparison between theory and scattering experiment. Macromolecules.

[30] Cosgrove T., Crowley T., Vincent B., Barnett K., Tadros T. (1981). The configuration of adsorbed polymers at the solid-solution interface. Faraday Symp. Chem. Soc..

